# Double J Stents and Reno–Ureteral Lithiasis: Dynamic Changes in Management during the COVID-19 Pandemic

**DOI:** 10.3390/life13112113

**Published:** 2023-10-25

**Authors:** Adrian Militaru, Catalin Andrei Bulai, Cosmin Ene, Razvan Ionut Popescu, Cosmin Cozma, Cristian Mares, Stefan Balacescu, Cristian Moldoveanu, Dragos Adrian Georgescu, Petrisor Aurelian Geavlete, Bogdan Florin Geavlete

**Affiliations:** 1 Faculty of Medicine, “Carol Davila” University of Medicine and Pharmacy, 8 Eroii Sanitari, 050474 Bucharest, Romania; 2 Department of Urology, “Saint John” Clinical Emergency Hospital, 13 Vitan-Barzesti, 042122 Bucharest, Romania; 3 Department of Urology, “Prof. Dr. Th. Burghele” Clinical Hospital, 20 Panduri, 050659 Bucharest, Romania

**Keywords:** urolithiasis, double J stent, ureteral obstruction, COVID-19

## Abstract

Purpose: To provide an evidence-based review of the use of ureteral stents in managing reno–ureteral lithiasis during the COVID-19 pandemic. Materials and Methods: A literature search was conducted between 2020 and 2023 using the PubMed and SCOPUS databases. As a part of the search query, we entered “ureteral stents” OR “double J stent” AND “renal colic” OR “ureteral obstruction” OR “reno-ureteral lithiasis” AND “COVID-19 Pandemic” OR “SARS-CoV-2 infection”. Results: Patients with lithiasis should be categorized into low priority, intermediate priority, high priority, and emergency under the COVID-19 pandemic scenario to manage their delay and save resources, including healthcare professionals, beds, and ventilators. However, immediate interventions are necessary for individuals at risk of life-threatening septic complications. During the COVID-19 pandemic, the feasibility of conducting or resuming elective activity depended on local circumstances, the accessibility of beds and ventilators, and the execution of screening protocols. If lithiasis surgery is delayed, consequences and increased effort will be inevitable. It is possible that teleconsultation could help guide these patients and cut down on unnecessary visits and exposure. Conclusions: COVID-19 has shifted treatment options for urinary stones, with ureteral stents being a safe, efficient, and cost-effective option for managing urolithiasis. Decompression is essential in emergency situations, while ureteral stents reduce the risk of infection and hospital visits.

## 1. Introduction

Urolithiasis, or kidney stone disease, is a common urological condition that often necessitates emergency medical attention. If this condition is left untreated, it may result in severe consequences [1]. There is an estimated prevalence of stone disease of 13% among men and 7% among women in the United States [2], while the reported percentage in Europe appears to be between five and nine percent [3]. According to recent data, urolithiasis prevalence is increasing worldwide due to various causes, such as dietary choices, climatic changes, social situations, and disease comorbidities [4]. The recurrence rate for this pathology is estimated to be around 50% at five years, reaching up to 75% at 20 years after the initial lithiasis episode [5,6].

The Chinese authorities reported the first COVID-19 case in Wuhan in December 2019. One month later, the pathology was identified as SARS Coronavirus 2 (SARS-CoV-2) [7,8]. Therefore, this virus spread throughout the world in a short time. On 11 March 2020, the World Health Organization (WHO) declared it a pandemic. The spread of the SARS-CoV-2 virus generated an unprecedented global crisis with a significant impact on health systems [9].

The 2019 Coronavirus disease (COVID-19) can cause acute respiratory distress syndrome (ARDS), which is often fatal [10,11]. As a result of the COVID-19 infection, elderly men with comorbidities are more prone to respiratory failure, and some patients rapidly progress to multi-organ dysfunction [12,13].

Renal colic caused by urolithiasis is an emergency with one of the highest emergency department (ED) addressability rates [14]. In some cases, especially in the first months of the pandemic, fear due to COVID-19’s high contagiousness led to a late presentation to the hospital. As a result, they reached more advanced stages of the disease or presented with very intense symptoms. This substantially decreased emergency room (ER) presentations [15,16].

This review aims to provide an evidence-based review of the use of ureteral stents in managing reno–ureteral lithiasis during the COVID-19 pandemic. In this article, we will refer to the recommendations of the European Association of Urology Guidelines Office Rapid Reaction Group regarding the treatment of urolithiasis in the COVID-19 era.

## 2. Materials and Methods

An extensive search was conducted in the PubMed and SCOPUS databases to identify literature published between 2020 and 2023. As a part of the search query, we entered “ureteral stents” OR “double J stent” AND “renal colic” OR “ureteral obstruction” OR “reno-ureteral lithiasis” AND “COVID-19 Pandemic” OR “SARS-CoV-2 infection”.

This review covers studies on the use of ureteral stents in the management of reno–ureteral lithiasis and ureteral obstruction. Considering the COVID-19 pandemic, we evaluated management approaches for urolithiasis, reviewed treatment criteria, and investigated variations in complications associated with ureteral stent insertion. Moreover, we tried to determine how newly developed guidelines reduced these complications during the current pandemic.

Due to the pandemic period, our goal was to identify potential care gaps. There were gaps in access to imaging and specialist services, which may have led to more complications during ureteral stent placement. Our review aimed to determine whether updated guidelines could reduce the risk of pandemic complications.

## 3. Results

### 3.1. The Use of Double J Stents in Urology before the Pandemic

Zimskind et al. described the use of ureteral catheters or double J stents for the first time in 1967 [17]. The devices are small, flexible tubes made of biocompatible materials, commonly silicone and polyurethane. They are inserted into the ureter to maintain its patency and allow urine to pass from the kidney to the bladder [18]. These stents range from 25 to 30 cm long and are inserted using a cystoscope on a metal guide under fluoroscopic guidance [19].

Since their discovery, they have been increasingly utilized in urological endoscopic surgery, becoming an essential part of daily practice [20]. Every year in the United States, approximately 92,000 stents are used for the treatment of obstructive ureteral pathology and reno–ureteral lithiasis. Stents are one of the most significant tools in a urologist’s arsenal [21,22].

It is estimated that more than 80% of ureteral stents present adverse reactions, resulting in severe pain and negatively affecting the patient’s quality of life. Often, this requires surgical intervention and ultimately increases healthcare costs [23,24]. Therefore, manufacturers have focused on developing biodegradable ureteral stents that significantly reduce the unpleasant side effects and complications linked with double j stents. The stents are made from biocompatible materials that reduce the risk of infection and other complications that can arise. In addition, they provide a much safer experience for the patient [25]. Until we benefit from their potential advantages, well-documented studies are still needed. However, this seems to be the path to follow in research on ureteral stents [26].

Ureteral stents were estimated to cost USD 359.9 million in 2018 and are predicted to reach USD 564.4 million in 2026. In addition to the direct costs of double J stents, some indirect costs are described. These costs include those linked to decreased quality of life, the drugs administered during stenting, the decrease in productivity at work, or even medical leaves [27,28,29]. In recent studies, silicone stents have been shown to be easier to tolerate in terms of stent-related symptoms [30,31,32].

In lithiasis pathology of the upper urinary system, double J stents are mainly used for drainage in case of ureteral obstruction, after ureteroscopy, or before extracorporeal shock wave lithotripsy (ESWL) [33]. Upper urinary drainage is mandatory in cases of infected hydronephrosis or sepsis but not necessary in all cases of ureteral obstruction. According to the guidelines of the European Association of Urology (EAU), double J stents have similar effectiveness to percutaneous nephrostomy in decompressing the urinary tract and in terms of recovery after sepsis [34,35,36]. Ureteral stents are mainly used (87.7%) to the detriment of percutaneous nephrostomy, according to a study conducted in the United States of America by Sammon et al. [37]. After the ureteroscopic treatment of uncomplicated reno–ureteral lithiasis, the EAU guide and that of the American Urological Association (AUA) state that the insertion of a urethral stent can be omitted [33,34,38], thus reducing urinary tract symptoms associated with stenting and reducing operating costs and time [39].

Although these tools are widely used, they also have disadvantages that reduce the quality of life [40]. Though many manufacturing techniques and materials have been evaluated, an ideal ureteral stent that provides optimal urinary drainage and is easy to mount, tolerable, and durable remains elusive [41,42,43,44]. They also have a whole series of complications, such as migration, obstruction, dysuria, lumbar pain, hematuria, urinary tract infection, encrustation, calcification, and even fragmentation [45,46]. Additional studies are needed to assess the need for perioperative stenting. Therefore, without clear indications, it remains up to each practitioner to assess the necessity of inserting a ureteral stent before or after an intervention; this is conducted by balancing benefits and complications.

### 3.2. The European Association of Urology Guidelines Office Rapid Reaction Group

Health systems worldwide faced an unprecedented situation because of the COVID-19 pandemic. With the rapid increase in COVID cases, the situation became more and more challenging to manage, even more so for surgical doctors. During this period, several changes were implemented in urolithiasis management. The EAU also convened a large group of experts to draw up suggestions that could help both patients and urologists; this was conducted to minimize impact and risk.

These guidelines focused on the urgent need to prioritize both treatments and surgeries based on the risk/benefit ratio. They also recommended using telemedicine where possible and ensuring the balance between the maximum use of resources and the safety of both patients and healthcare workers. Color codes were used according to risk stratification. Depending on their severity, these were classified as low priority, which could be postponed for up to 6 months. They were also classified into intermediate priority, high priority, and emergency, which cannot be postponed for more than 24 h. In times of pandemic, it is recommended to implement standardized surgical techniques to cut down on operating room time and postoperative complications. For those reasons, all surgical procedures should be performed by qualified surgeons who have completed the learning curve. Implementing updated technology and conducting specific research on evolving technologies should be delayed until a pandemic emergency passes [47,48].

### 3.3. Stone-Related Emergencies in the Event of the Pandemic

Most studies show a decrease in urological emergencies referred to ERs for consultation and specialized treatment; this seems to be due to restrictions imposed during the lockdown period and fear of contagion. As a result, there was an increase in the number of patients with more severe conditions who needed hospital admission and specialized care. Healthcare facilities had to adapt quickly to the changing situation. They put protocols in place to ensure patient safety. Novara et al., in a study carried out in Italy, the first European country severely hit by the COVID pandemic, showed that the number of urological emergencies addressed to ED in one week of 2020 decreased by 55% compared to the same period in 2019. At the same time, he observed an increase in the number of endoscopic interventions (JJ stent insertion or ureteroscopy) for lithiasis pathology in 2020 compared to 2019 due to the desire to treat symptoms and avoid urosepsis. In this way, he claims to have reduced the number of hospitalizations and implicitly reduced the risk of contagion [49].

A multicenter analysis also carried out in Italy by Antonucci et al., shows a reduction in the number of hospitalizations for renal colic by 48.8% in the period March–April 2020 compared to the same period of 2019, with differences varying between 30 and 69.3% depending on the center [50].

A study carried out in Poland by Kaczmarek also showed a decrease in the hospitalization rate in the Urology department in the October–December period of 2020 compared to the same period of 2019. The number of hospitalizations decreased by 35.9% in comparison to the first peak of the pandemic in Poland and by 56.41% compared to the second peak [51].

Steinberg and colleagues also saw a 38% and 44% decrease in ED visits for stone illness at their private academic and county hospitals in Dallas, USA, respectively [52].

### 3.4. The Use of Double J Stents in Urological Emergencies during the Pandemic

Considering the limited availability of anesthesiologists and ventilators during the COVID-19 pandemic, it is preferable to perform procedures under local anesthesia, even in the management of urgent urological conditions. For instance, it promotes the use of ureteral stents in upper urinary tract obstruction treatment since they simplify at-home care. Whenever possible, the cause of the obstruction should be addressed according to the resources readily available in the area. However, percutaneous nephrostomy or ureteral stenting under local anesthesia is advised to drain the upper urinary tract without anesthesiology assistance [53].

The COVID-19 practice pattern for urolithiasis has significantly changed. Based on the EULIS Collaborative Research Group, following the COVID-19 pandemic, urolithiasis practice patterns were evaluated through a large survey involving sixty physicians whose primary area of expertise is urinary stones. According to the poll, 49% of specialists reported that their regular therapeutic practices had changed by more than 90%. During the crisis, 72.3% used telemedicine. A total of 89.4% of respondents reported that they were more likely to plan interim collection system draining followed by an elective intervention for COVID-19 emergency patients. However, 10.6% of them continued to undergo final stone surgery treatment. Among the respondents, their elective surgical treatment approach changed as follows: 55.3% at a rate of 90–100% and 39.8% at a rate of 75–89% after COVID-19. Even so, 6.4% of them remained the same as they had been before the outbreak [48].

It took roughly 21 days for various hospitals to implement COVID-19 adjustments and interventions for kidney stones. While the rate of definitive treatments like ureteroscopy (URS), retrograde intrarenal surgery (RIRS), and percutaneous nephrolithotomy (PCNL) decreased from 60.8 to 19%, the rate of conservative approaches, such as nephrostomy tube (NPT) insertion, double JJ stent placement, or extraction, increased significantly from 38.2 to 81% [48].

There is still a question about the definitive treatment for the COVID-19 pandemic. Some urologists chose active stone treatment over interim drainage, except when an infection was present or staged treatment was anticipated; this was to minimize ED visits. Others would rather wait until the COVID-19 pandemic was over before beginning operations to treat urinary stones, merely performing temporary drainage when necessary [50,54]. When the obstruction is accompanied by infection and fever, we should use an indwelling JJ stent or NPT to temporarily drain the collecting system before beginning, if possible, definitive treatment [55,56].

How to treat patients who already had ureteral stents for severe urolithiasis prior to the COVID-19 epidemic is another issue of concern. The infection relayed to the urinary stent can cause serious morbidity, including acute pyelonephritis, bacteremia, urosepsis, and even death. This subset of patients should be given significant consideration to prevent a lengthy wait. Bearing in mind that most ureteral stents can be left in place for up to 6–12 months, the length of time the stent will be in place should be a consideration in the prioritization process. Even though there is currently insufficient evidence to support antibiotic prophylaxis for patients with indwelling stents, considering at least some pulse antibiotic therapy to lower the risk of urosepsis and the ensuing need for a mechanical ventilator could be worthwhile given the likely delays in surgery [57]. Additionally, endourologists must be ready to handle more challenging situations for patients whose surgical procedures are delayed due to lower priority. Waiting lists should also increase. To check on the status of their stones, these patients should be regularly followed up with phone calls [55].

Decompression is recommended for obstructed or infected renal and ureteral stones. There is an agreement, however, that non-obstructed kidney stone treatment can be postponed for several months. Still, it is crucial to remember that patients with symptomatic ureteral/renal stones and those with stents should be given priority care [47,58,59].

For patients with proven or suspected COVID-19, Proietii et al. recommend urgent endourological stone surgery must be performed differently, and these patients must be handled in a special operating theater with a negative pressure environment (Table 1). To shorten the surgical time, ureteral stent positioning or percutaneous NPT should be chosen over URS and stone fragmentation. Spinal anesthesia should be used to prevent ventilation and aerosol production [55].

Carneiro et al. [60] recommended that COVID-19-negative patients needing urgent intervention for ureteric stones undergo definitive lithotripsy whenever possible and well tolerated, with the postoperative placement of a stent on a string, instead of only drainage, in contrast to the general trends of delaying definitive treatment during the pandemic. They claimed that this approach would result in effective treatment and fewer hospital visits throughout the pandemic.

A variety of procedures and strategies were suggested to lower the likelihood of hospital admissions during the epidemic. During the pandemic surge, several triage algorithms recommended delaying the final treatment of non-urgent nephrolithiasis for longer than 12 weeks. Some examples included patients needing PCNLs, those with stents or NPT, and those with asymptomatic stones [53,55,56,58]. Replacement of ureteral stents and NPT was delayed for up to 6 months [53]. Additionally, it was advised to postpone treatments in patients with indwelling stents because research shows that most stents may be successfully removed with outpatient surgery after being kept in place for up to 6–12 months [61]. However, one should be mindful of the hazards involved in NPT or indwelling stents for long periods. Prior to COVID-19, prospective research found that the length of time an indwelling stent was left in place was a major risk factor for post-URS sepsis [62]. Based on these results, the authors suggested that stent insertion be approached cautiously and that, if necessary, definitive URS be carried out within a month.

The possibility of skipping post-procedure stent implantation was also investigated during the pandemic while considering the risk of long-indwelling ureteral stents. According to Kachroo et al. [63], their rate of stent omission increased from 12% to 66% over the COVID-19 era. Likely due to COVID-19, their frequency of abandoning stents with strings increased from 7% to 16%. Interestingly, the authors’ stent policy adjustments did not cause any issues that necessitated ED visits. These findings support the recommendation to keep the stent string because it would enable the patient to remove the stent without a doctor’s visit, saving time and money. The use of a silicone stent rather than a polyurethane stent to lower the danger of encrustation is one of the other ways investigated to lessen concerns regarding the long-term installation of indwelling ureteral stents [62].

**Table 1:** Summary of publication on urolithiasis regarding endourological procedures during the COVID-19 Pandemic.

No	Authors	Title	Year	Type of Study	Conclusions/Recommendations
1	Ribal et al. [47]	European Association of Urology Guidelines Office Rapid Reaction Group: An Organization-wide Collaborative Effort to Adapt the European Association of Urology Guidelines Recommendations to the Coronavirus Disease 2019 Era.	2020		- Emergency stenting of the collector system with double J stent or nephrostomy in cases with infected hydronephrosis.
2	Antonucci et al. [50]	The impact of COVID-19 outbreak on urolithiasis emergency department admissions, hospitalizations, and clinical management in central Italy: a multicentric analysis.	2020	Multicenter study	- In Rome, there has been a decrease in hospitalizations for urolithiasis and an increase in cases presented with a higher rate of complications.
3	Ficarra et al. [53]	Urology practice during the COVID-19 pandemic.	2020	Short communication	- Upper urinary tract obstruction or infection represents emergencies, and inserting a nephrostomy tube or a ureteral stent under local anesthesia is recommended.
4	Proietti et al. [55]	Endourological Stone Management in the Era of the COVID-19.	2020	Editorial	- Renal colic: managed conservatively; - Non-obstructing renal and ureteral stone: delay; - Stent removal: delay; - Obstructed kidney/infection: double J stent or nephrostomy.
5	Metzler et al. [56]	Stone Care Triage During COVID-19 at the University of Washington.	2020	Commentary	- Ureteral stone with obstruction and infection: double J stent or nephrostomy; - Consider stentless to avoid readmission in the hospital.
6	Goldman [58]	Recommendations for Tiered Stratification of Urological Surgery Urgency in the COVID-19 Era.	2020	Editorial	- Obstruction of the kidney/infection: drainage.
7	Carneiro et al. [60]	Impact of the COVID-19 Pandemic on the Urologist’s Clinical Practice in Brazil: a management guideline proposal for low- and middle-income countries during the crisis period.	2020	Letter to the Editor	- Patients who already have a double J stent can be timed as long as possible; - Patients with urolithiasis, except for emergencies, should be postponed; - Those with urinary obstruction and infection need drainage.
8	Stensland et al. [61]	Considerations in the Triage of Urologic Surgeries During the COVID-19 Pandemic.	2020	Platinum opinion	- In certain situations, stents or nephrostomy tubes can also be placed at the bedside or under local anesthesia, sparing a ventilator.
9	Raheem et al. [64]	Impact of COVID-19 on endourology surgical practice in Saudi Arabia: A national multicenter study.	2021	Multicenter study	- Interventions like PNL and SWL have been almost suspended; - The rate of telephone consultations increased in most hospitals.
10	Silva et al. [65]	COVID-19 pandemic impact on clinical outcomes of patients with obstructive pyelonephritis.	2021	Original article	- During the COVID-19 pandemic, individuals with acute obstructive pyelonephritis presented to the emergency room with a longer delay between symptoms onset and greater illness severity and a longer length of hospitalization.
11	Mazzon et al. [66]	The effect of COVID-19 outbreak on urological procedures for urinary stones: data from three high-volume centers in China.	2022	Multicenter study	- There was an upward trend in the number of complicated cases as the surgical volume decreased.

## 4. Discussions

As a result of the COVID-19 pandemic, the healthcare sector has been directly affected. In this sense, urology has also suffered from this epidemic. These impacts have played an essential role in the restructuring of both elective and emergency urological surgical procedures. Studies examining COVID-19’s effects on urology are published, and they contend that these updated guidelines have forced significant changes in how urological patients are managed [53].

The reorganization of resources and the prioritization of medical care aimed to ensure that patients with high-risk conditions received continuous, appropriate, and timely assessment and management while limiting unnecessary danger and strain caused by circumstances in which it is safe to delay treatment. In this regard, a practical check of the healthcare infrastructure was necessary based on the availability of health system resources, such as beds in intensive care units, ventilators, personal protective equipment, COVID-19 testing, and specialists in the healthcare field. Sound surgical judgment can lessen the strain on healthcare systems. Whenever it is clinically appropriate for the patient, nonoperative management should serve as an option for treatment. In addition, these considerations may reduce the number of people working on a team and increase local healthcare providers’ ability to respond to an emergency.

There were widespread requests for people to remain indoors, and public health authorities mandated that elective surgical procedures be delayed until further notice. It suggested that public health orders aimed at preventing COVID-19 spread in the immediate area, such as social isolation and lockdowns, were effective. These recommendations should be updated as the situation develops, including attempts to return to the original normal and the potential for fresh infection waves [59].

Seven out of thirty-four patients (20.5%) who underwent elective procedures in Wuhan died, as reported by Lei et al. [67]. These individuals were in the latent or hospital-acquired infection phase when they presented, making them asymptomatic carriers.

Even though urinary stones are generally harmless, they can cause severe complications if not treated promptly. These complications include urosepsis, deterioration of renal function, prolonged pain, and the need for repeated access to emergency care services, among others. Endoscopic procedures were also significantly affected. Due to the outbreak, patients were at risk, creating an overload on healthcare systems already experiencing difficulties providing adequate care. A delay in treatment may also result in reduced quality of life for patients with urinary stones. A stent may cause extended irritative symptoms, anxiety, or stone-related symptoms [57].

It is essential to emphasize the necessity of treating individuals with obstructed and infected stones, with rapid decompression being the treatment of choice whenever possible. A telemedicine program to monitor clinical progression is justified in patients with risk factors such as a pre-existing indwelling ureteral stent, symptomatic, recurring emergency visits, solitary kidney, and bilateral ureteral calculi [59]. During the current digital era, social media has become one of the most popular ways to find medical advice on various medical conditions, especially during the recent COVID-19 pandemic. Consequently, this can be a double-edged sword, as misinformation can also be spread through online platforms, leading people to make decisions that are not in their health's best interest. The source of any advice received on social media should be verified, and a medical professional should be consulted if necessary [68,69].

Naspro et al. [70] found that urological surgery volumes dropped by 30% in the first 15 days after the outbreak began. The same authors predicted everything would stop working on March 19, 2020. The number of occupied hospital beds among COVID-19 patients indicated as much. The lack of anesthesiologists, operating rooms, and ventilators necessary for critically ill patients also severely hampered the ability to provide emergency urological surgery to COVID-19 patients. In addition, Novara G et al. found that the number of urgent consultations in Italy dropped by 55%, with a peak reduction of 64% in regions hit hardest by the COVID-19 pandemic [49].

In Saudi Arabia, Bin Hamri et al. found the same thing [64]. Overall, the number of outpatient clinic visits (of which 90.8% were conducted via telephone consultations) and elective procedures performed by doctors decreased by 78% and 34%, respectively, as noted by the authors. There was also a 9.3% decrease in emergency procedures.

Urinary stones pose a significant risk to patients if not treated properly. Galiabovitch et al. [71] found that stones account for 10% of surgical mortality. Furthermore, 49.5% of all deaths are caused by urosepsis. The same scientists also found that subpar or delayed infection care accounted for 39% of their findings. Many people reported delaying medical care or using less-than-ideal treatments because of the pandemic. These findings highlight COVID-19’s potential impact on patients with kidney stones. Knowing the instances of kidney failure, ureteral strictures, and infections that might arise from urolithiasis treatment delays is difficult.

Delays in treating renal obstruction complications have contributed to more severe outcomes in the medical literature. Before the pandemic, a longer delay between the onset of symptoms and the proper intervention for blocking urosepsis was associated with an increased risk of septic shock [72,73]. Patients with obstructive pyelonephritis were more likely to have severe clinical conditions, such as systemic inflammatory response syndrome (57%) and perirenal abscesses (13% versus 0%) and spend longer in the hospital (mean 7.6 days versus 3.8 days) during the COVID-19 pandemic, as demonstrated by Meller et al. [65]. Researchers concluded that rigorous procedures implemented in Brazil and the corresponding unwillingness of the population to seek hospital aid contributed to the delay in treatment. Mazzon et al. also saw a decline in emergency interventions but no rise in potentially fatal conditions [66].

Gul and colleagues discovered that serum creatinine levels and white blood cell counts at hospital admission were significantly higher during the COVID period. Additionally, grade 3 and grade 4 hydronephrosis also increased during this time. These findings reflect the higher incidence of complex ureteral stone disease during COVID-19 restrictions [74].

Because removal is usually easy for stents with an indwelling period of 6 to 12 months, Stenzel et al. encouraged delaying most surgeries for indwelling ureteral stent removal [61]. Instead, to prevent stent encrustation, recurrent infections, and bothersome stent symptoms that necessitate ER visits or hospital admission, as well as to reduce the risk of stents being retained/forgotten, Katz et al. advised ureteral stent removal as an urgent office-based procedure [75]. Rarely, infections linked to ureteral stents can result in life-threatening conditions such as acute pyelonephritis, bacteremia, urosepsis, and even death. During COVID-19, stentless treatments were therefore recommended after successful operations. We also considered employing urethral stents with strings that can be removed without hospitalization [55,56,60].

## 5. Conclusions

During the COVID-19 epidemic, significant shifts occurred in the available options for treating urinary stones. Obstructed reno–ureteral stones or infected ones should be treated as an emergency by decompression. Ureteral stents were a safe, efficient, and cost-effective procedure for urolithiasis during the COVID-19 pandemic. In addition, they reduce the risk of infection and hospital visits. Therefore, it was a valuable option in urolithiasis treatment during the pandemic.

## Data Availability

Not applicable.
